# Changes in Smoking Patterns and Cervical Cancer Risk: Preventive Implications from a Nationwide Japanese Cohort

**DOI:** 10.3390/healthcare13222852

**Published:** 2025-11-10

**Authors:** Yun Jeong Lee, Sun Yeup Kim, Nang Kyeong Lee, Seung Won Lee

**Affiliations:** 1 Department of Metabiohealth, Sungkyun Convergence Institute, Sungkyunkwan University, Suwon 16419, Republic of Korea; aristata52@g.skku.edu; 2 Department of Medical AI, Sungkyunkwan University School of Medicine, Suwon 16419, Republic of Korea; paul5506@g.skku.edu; 3 Department of Precision Medicine, Sungkyunkwan University School of Medicine, Suwon 16419, Republic of Korea; skdrud3717@skku.edu; 4 Department of Artificial Intelligence, Sungkyunkwan University, Suwon 16419, Republic of Korea; 5 Personalized Cancer Immunotherapy Research Center, Sungkyunkwan University School of Medicine, Suwon 16419, Republic of Korea; 6 Department of Family Medicine, Kangbuk Samsung Hospital, Sungkyunkwan University School of Medicine, 29 Saemunan-ro, Jongno-gu, Seoul 03181, Republic of Korea

**Keywords:** cervical cancer, smoking pattern changes, screening, cancer prevention, epidemiology, risk stratification, nationwide cohort study

## Abstract

Background/Objectives: Smoking is an established cofactor for cervical carcinogenesis, but evidence on how Smoking Pattern Changes around cohort entry relate to risk in Japan is limited. We quantified cervical cancer risk by baseline smoking status and by changes between two routine health checkups in a nationwide cohort. Methods: We used the Japan Medical Data Center claims–checkup database between January 2005 and July 2022. Women with ≥2 pre-index checkups were included; the index date was the second checkup. Self-reported smoking at each visit defined never, former (quit), new (initiated), and current (persistent) smokers; checkup pairs >36 months apart were excluded. Incident cervical cancer required ICD-10 C53 plus cancer-directed treatment (surgery, radiotherapy, or systemic antineoplastic therapy). Multivariable Cox models estimated hazard ratios (HRs) with 95% CIs, adjusting for age, BMI, alcohol, exercise, hypertension, diabetes, cerebrovascular and cardiovascular disease, and cholangitis. Results: Among 1,330,797 women, incidence rates (per 100,000 person-years) were 151.4 in never smokers and 244.9 in ever smokers. Ever versus never smoking was associated with higher risk (HR 1.53, 95% CI 1.43–1.62). A graded risk was observed across Smoking Pattern Change categories versus never: former HR 1.44 (1.15–1.79), new HR 1.51 (1.20–1.90), current HR 1.54 (1.44–1.64). By age, HRs were 1.58 (1.47–1.70) for <50 years and 1.35 (1.17–1.55) for 50–64 years; ≥65 years was not statistically significant (HR 0.69, 0.30–1.59). Conclusions: Smoking was associated with substantially higher cervical cancer risk, with a clear risk gradient from former to new to current smoking. The rapid elevation in new smokers and residual risk after quitting support integrating proactive cessation and initiation prevention into risk-stratified screening and routine health-check programs in Japan.

## 1. Introduction

Cervical cancer presents a unique challenge in modern oncology as one of the most preventable and screenable malignancies. This has culminated in the World Health Organization’s (WHO) Global Strategy to Accelerate the Elimination of Cervical Cancer, built upon HPV vaccination and effective screening [1]. While high-income countries have made progress, the global burden remains substantial [2,3]. This is particularly evident in East Asia, where countries like Japan face persistent challenges. In 2022 alone, Japan reported approximately 11,700 new cervical cancer cases and 2900 deaths, underscoring the urgent need for optimized prevention strategies [3]. This high incidence is largely attributable to a historically low HPV vaccination rate, which plummeted from over 70% to less than 1% for several years following unsubstantiated safety concerns, creating a cohort of susceptible young women [4].

While HPV is the principal cause, modifiable cofactors that accelerate carcinogenesis remain critical targets; among these, smoking is especially important [5]. A robust biological rationale supports targeting smoking for cervical cancer prevention. Tobacco-specific carcinogens directly damage the cervical epithelium, induce local immunosuppression, and ultimately reduce HPV clearance, prolonging the window for viral persistence and progression [6,7,8,9]. Beyond viral persistence, smoking independently increases the risk of high-grade cervical intraepithelial neoplasia and cervical cancer, even after accounting for HPV infection status and other confounders [10,11].

Despite this compelling evidence, a critical knowledge gap remains within the Japanese context. Most prior evidence is cross-sectional, establishing only a static link between smoking and disease [12]. To our knowledge, and as highlighted by a recent meta-analysis of Japanese studies which noted a lack of robust evidence on the effects of smoking cessation [11], no longitudinal study has specifically analyzed how dynamic changes in smoking patterns—such as cessation [13], relapse, or continued smoking—impact the subsequent risk of cervical cancer in this population. This is a significant omission, as understanding the effects of such behavioral changes is crucial for effective public health messaging and intervention.

Furthermore, accurately capturing these behavioral changes is methodologically challenging within the Japanese context. The study period (2005–2022) witnessed a dynamic tobacco landscape, marked by the diversification from traditional cigarettes to include the rapid proliferation of heated tobacco products (HTPs, e.g., IQOS) and e-cigarettes [14]. Concurrently, regulations shifted from an emphasis on social ‘manners’ to binding ‘rules’ [15]. This evolving context, characterized by product switching and dual-use, likely introduces variability and potential bias (i.e., measurement issues) into self-reported smoking status over time [14]. This underscores the necessity of an analytic approach that uses repeated measurements to operationally define smoking status, rather than relying on a single assessment.

Therefore, the primary objective of this study was to determine how changes in smoking status between two consecutive health checkups affect cervical cancer incidence in Japan. By analyzing a large nationwide cohort, we aim to provide actionable evidence for developing risk-stratified screening strategies and integrating cessation support into the national cancer elimination agenda [16,17].

## 2. Materials and Methods

### 2.1. Ethics Statement and Data Source

This retrospective cohort study was conducted using a de-identified dataset from the Japan Medical Data Center (JMDC) database. The study protocol adhered to the tenets of the Declaration of Helsinki and was granted an exemption from full board review by the Institutional Review Board (IRB) of Sungkyunkwan University (Exemption No. 2025-09-070). The IRB also waived the requirement for informed consent due to the secondary use of a fully anonymized database.

The JMDC database is one of Japan’s largest claims databases, integrating medical and pharmacy claims (diagnoses, procedures, and prescriptions) with annual health check-up records from multiple company-based health insurance societies. Our initial study population included approximately 5 million individuals who underwent at least two health checkups between January 2005 and July 2022. It should be noted that the JMDC population primarily consists of company employees and their dependents, which may limit the generalizability of our findings to elderly or unemployed populations [18].

### 2.2. Study Population

From the JMDC database, we first identified 5,345,907 individuals who had at least two recorded health checkups. The index date, which serves as the starting point for follow-up, was defined as the date of the second health checkup. This approach was chosen to establish a clear baseline and observe changes in health status or behaviors between the two checkups, ensuring that our analysis captured a dynamic health profile rather than a single static point in time.

We then applied several exclusion criteria to refine the cohort. First, we excluded individuals with a prior diagnosis of any cancer (ICD-10 C codes) before the index date (*n* = 466,513). This step was critical to prevent confounding from pre-existing malignancies and to ensure that cervical cancer could be analyzed as the primary outcome of interest. We also excluded men (*n* = 3,277,486) and individuals with an interval greater than three years between their first and second checkups (*n* = 51,084).

Finally, we excluded participants with an indeterminate smoking classification and those diagnosed with cervical cancer on the index date itself (*n* = 220,027). The latter group, corresponding to individuals with zero follow-up days, was excluded to ensure a clear temporal sequence between the baseline assessment and a subsequent cancer diagnosis. This step guarantees that the analysis focuses strictly on incident cases occurring after the baseline was established.

**Figure 1:**
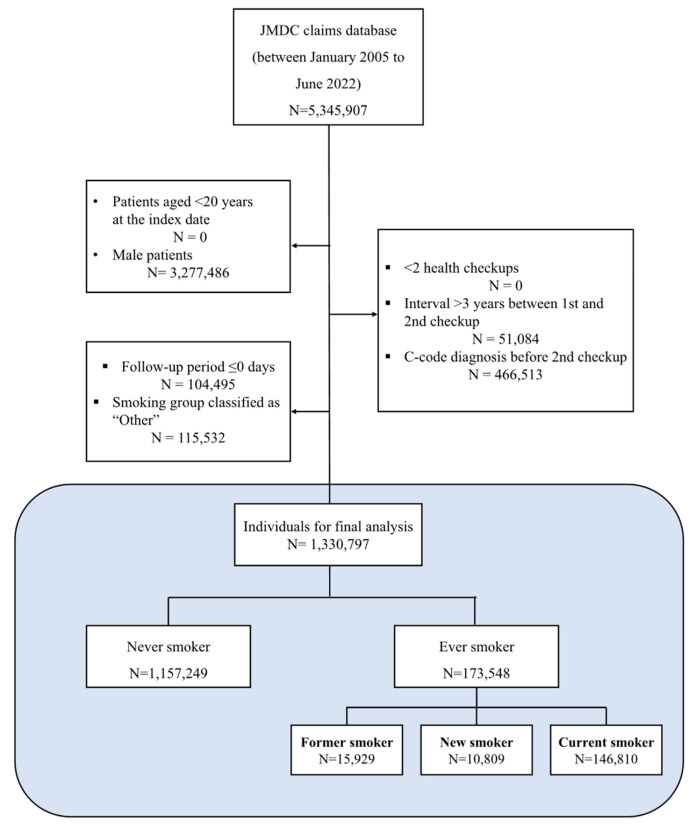
Selection of the study cohort from the Japan Medical Data Center (JMDC) database.

After applying these criteria, the final analytic cohort comprised 1,330,797 women. The detailed cohort assembly process is illustrated in Figure 1.

### 2.3. Definitions of Smoking Exposure

Smoking exposure was defined from the two routine health checkups immediately preceding cohort entry; the index date was the date of the second (later) checkup, as this represents the most up-to-date assessment of smoking status and other health characteristics immediately prior to the start of follow-up [19,20]. We required the inter-checkup interval to be ≤36 months, chosen to align with the typical 1–2-year periodicity of national health checkups in Japan while allowing a pragmatic window to capture changes in smoking behavior [21,22]; pairs exceeding this interval were ineligible. When three or more pre-index checkups were available, we used the two consecutive visits closest to the index date. At each checkup, current smoking was self-reported to the item “Do you currently smoke?” with response options yes/no; “yes” indicates smoking at the time of the checkup (current smoker), whereas “no” indicates not currently smoking at that visit (which may include never- and former-smoker status). Combining responses across the two pre-index visits, participants were classified into four mutually exclusive categories: never (non-smoker at both checkups), former (smoker at the first; non-smoker at the second), new (non-smoker at the first; smoker at the second), and current (smoker at both). It is important to note that the “New” category is an operational definition representing participants who newly reported smoking during the study’s observation window. This definition is not intended to be synonymous with ‘lifetime smoking initiation.’ This “New” group may potentially include individuals who relapsed after a period of long-term cessation, those reporting HTP or e-cigarette use for the first time (i.e., product switching), or statistical artifacts arising from left-censoring of a participant’s earlier smoking history. This baseline classification, based on changes in smoking behavior, was designed to capture smoking initiation, cessation, and persistence, an approach consistent with longitudinal smoking research [23,24]. Participants with missing or indeterminate (“other”) smoking responses at either visit were excluded from the primary analysis.

### 2.4. Definition of Outcome

The primary outcome was incident cervical cancer, identified using claims data. An incident case was defined as having at least one claim with an ICD-10 code for cervical cancer (C53) combined with a claim for a cancer-specific treatment, which included relevant surgical procedures such as cervical conization or hysterectomy, radiotherapy, or the administration of an antineoplastic agent. This combined diagnosis-and-treatment definition is consistent with previously validated algorithms [25,26]. The event date was the earliest qualifying claim date; when only the month was available, it was imputed as the 15th to maintain a consistent day-level timescale. Individuals with a history of any malignant neoplasm (ICD-10 C codes) before cohort entry were excluded.

Baseline covariates were obtained from health-check and claims data at or before the index date. These included age (continuous; categorized as <50, 50–64, ≥65), BMI (categorized as obese, overweight, normal, or underweight), drinking habit (daily, sometimes, or none), regular exercise (yes or no), and comorbidities (hypertension, diabetes mellitus, cerebrovascular disease, cardiovascular disease, and cholangitis). The selection and categorization of covariates were informed by established methodologies from prior claims-based studies in East Asian populations [26,27,28].

### 2.5. Follow-Up and Censoring

Follow-up for all participants began on the date of the second health checkup (index date), which was set as the baseline to capture changes in smoking status. Follow-up ended on the date of incident cervical cancer diagnosis or the last observation date, whichever came first. To ensure consistency, all analyses used a day-level timescale. However, the observation end dates in the administrative claims database were only available at the calendar-month level. To reconcile this difference in data precision, these dates were imputed to the 15th of the month. This midpoint imputation is a standard method used to minimize the systematic bias that would arise from selecting the first or last day of the month, thus allowing for a more accurate day-level calculation.

### 2.6. Statistical Analysis

Baseline characteristics were summarized by smoking category using counts and percentages for categorical variables and means with standard deviations (SDs) for continuous variables. Time-to-event associations were evaluated with Cox proportional hazards models to estimate hazard ratios (HRs) and 95% confidence intervals (CIs). We fit three sequential models: Model 1, univariable; Model 2, adjusted for age; and Model 3, a multivariable model adjusted for age, BMI category, drinking habit, regular exercise, hypertension, diabetes mellitus, cerebrovascular disease, cardiovascular disease, and cholangitis. Time-varying HPV vaccination and smoking-cessation treatment were not modeled because consistent capture and coding were not feasible across years and facilities in JMDC. The proportional hazards assumption was checked for all models. Kaplan–Meier curves were used to visualize the cumulative incidence by smoking category, and differences between groups were assessed using the log-rank test. Missing data were handled by complete-case analysis. All analyses were performed using SAS version 9.4 and R version 4.3, and a two-sided *p*-value < 0.05 was considered statistically significant.

## 3. Results

### 3.1. Baseline Characteristics of Study Participants by Smoking Status 

Among a total of 1,330,797 women, 173,548 (13.0%) were ever smokers and 1,157,249 (87.0%) were never smokers. Ever smokers were composed of former smokers (*n* = 15,929), new smokers (*n* = 10,809), and current smokers (*n* = 146,810). At baseline, the characteristics of the participants showed statistically significant differences across smoking categories (*p* < 0.001).

The baseline characteristics of the study participants according to smoking category are summarized in Table 1.

Specifically, the mean age of never smokers was 43.7 (±11.7) years. Among ever smokers, current smokers were the oldest at 44.4 (±10.6) years, followed by former smokers at 41.3 (±11.5) years, while the new smoker group was the youngest at 39.0 (±11.6) years. Regarding body mass index (BMI), the proportion of normal weight was highest among never smokers (68.66%), Conversely, the proportions of underweight (16.14%), overweight (12~14%), and obese (5.42%) individuals were all relatively higher among current smokers.

In terms of lifestyle, the rate of daily alcohol consumption was highest in the current smoker group. There was also a difference in the proportion of missing responses for drinking habits, which was higher among former smokers (9.44%) and new smokers (8.53%) compared to never smokers (6.54%). The proportion of participants who reported engaging in regular exercise was notably higher among ever smoker groups (former 13.20%, new 14.34%, current 13.40%) than in the never smoker group (8.40%). As for comorbidities, the prevalence of diabetes mellitus (13.34%) and cardiovascular disease (4.38%) was high in former smokers, while the rates of hypertension (8.75%) and diabetes (11.29%) were also high among current smokers. The prevalence of cholangitis was very low across all groups. Overall, these findings suggest a higher cardiometabolic burden among ever smokers, particularly in former and current smokers. The mean follow-up duration was longest for never smokers at 3.14 (±2.53) years.

### 3.2. Cervical Cancer Incidence by Smoking Status

**Figure 2:**
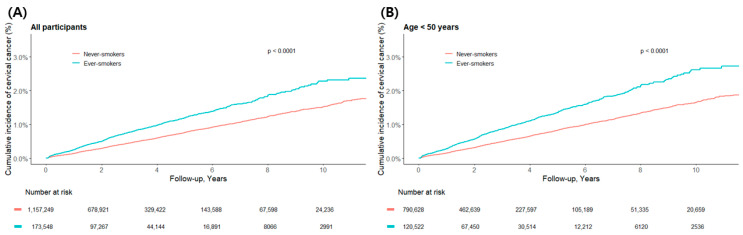

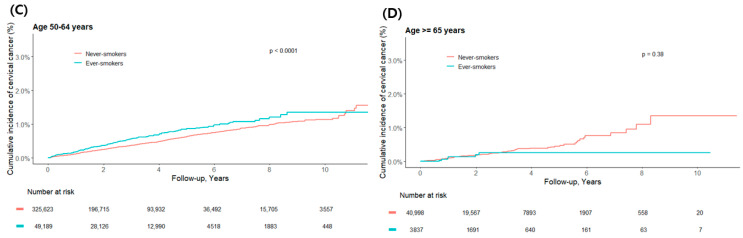
Cumulative incidence of cervical cancer by smoking status. The cumulative incidence of cervical cancer among ever-smokers and never-smokers is shown for (**A**) all participants, and stratified by age groups: (**B**) <50 years, (**C**) 50–64 years, and (**D**) ≥65 years. The number of individuals at risk is listed below each panel. *p*-values were derived from log-rank tests comparing the two groups within each stratum.

Kaplan–Meier analysis showed that the cumulative incidence curve for cervical cancer in ‘Ever smokers’ was significantly higher than that for ‘Never smokers’ (log-rank *p* < 0.0001) (Figure 2A).

This visual trend is clearly supported by the quantitative data presented in Table 2. During the follow-up period, 5481 cases of cervical cancer occurred in the never-smoker group, and 1247 cases occurred in the ever-smoker group. When converted to incidence rates, a notable difference was observed: 151.4 per 100,000 person-years for never-smokers compared to 244.9 for ever-smokers. After adjusting for other variables in the final model (Model 3), ever-smokers had a 1.53-fold higher risk of developing cervical cancer than never-smokers (HR: 1.53, 95% CI: 1.43–1.62).

Similar trends were observed in the age-stratified subgroup analyses. In both the <50 years and 50–64 years age groups, the cumulative incidence curves for ever-smokers were significantly higher than for never-smokers (Figure 2B,C). According to Table 2, in the <50 years group, cancer occurred in 4130 never-smokers and 993 ever-smokers, with smokers having a 1.58-fold higher risk (HR: 1.58, 95% CI: 1.47–1.70). In the 50–64 years group, there were 1254 cases among never-smokers and 248 among ever-smokers, with smokers having a 1.35-fold increased risk (HR: 1.35, 95% CI: 1.17–1.55).

However, in the ≥65 years age group, no significant difference was observed in the cumulative incidence (Figure 2D), and the adjusted hazard ratio in Table 2 also failed to reach statistical significance (HR: 0.69, 95% CI: 0.30–1.59). This can be attributed to the limited statistical power due to the very small number of events in this age group, with 97 cases among never-smokers and only 6 cases among ever-smokers.

Hazard ratios (HRs) with 95% confidence intervals (CIs) were estimated using Cox proportional hazards models, with ‘Never smoker’ as the reference category. Model ^1^ is univariable; Model ^2^ is adjusted for age; Model ^3^ is the multivariable model, adjusted for age, BMI, alcohol consumption, regular exercise, and comorbidities (hypertension, diabetes mellitus, cerebrovascular disease, cardiovascular disease, and cholangitis). Incidence rates are presented per 100,000 person-years with 95% confidence intervals (CIs) were calculated. Smoking categories (former, new, current, never) were defined based on two consecutive health checkups.

### 3.3. Association Between Smoking Exposure and Outcome

**Figure 3:**
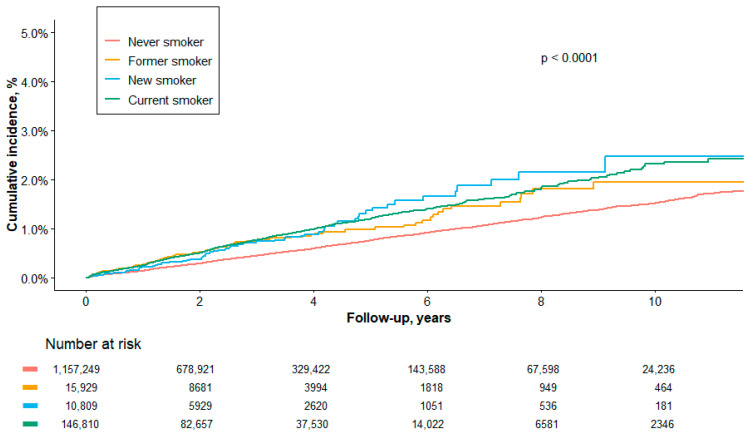
Cumulative incidence of cervical cancer by detailed smoking category.

The relationship between dynamic changes in smoking status and cervical cancer risk is illustrated in the Kaplan–Meier curves in Figure 3. All groups with smoking exposure—former, new, and current smokers—had a significantly higher cumulative incidence of cervical cancer compared to never-smokers (log-rank *p* < 0.0001).

The quantitative analysis in Table 3 revealed a clear gradient of risk across these groups. The incidence rate per 100,000 person-years was lowest among never-smokers (151.4) and was progressively higher for former smokers (229.1), new smokers (241.4), and current smokers (247.0). After adjusting for covariates (Model 3), the risk remained significantly elevated for all groups compared to never-smokers. The hazard ratio (HR) was 1.44 (95% CI: 1.15–1.79) for former smokers, 1.51 (95% CI: 1.20–1.90) for new smokers, and highest for current smokers at 1.54 (95% CI: 1.44–1.64).

Taken together, these findings demonstrate that continued smoking is a potent risk factor for cervical cancer. The persistently elevated risk, even among those who have quit, underscores the critical importance of smoking cessation in preventing this disease.

The cumulative incidence of cervical cancer differed significantly among the smoking groups (log-rank *p* < 0.0001). As illustrated in Figure 3, there was a graded, exposure-dependent risk, with the highest incidence observed in current smokers, followed by new and former smokers, and the lowest in never smokers.

Kaplan–Meier curves for never, former, new, and current smokers. The y-axis shows cumulative incidence (%), and the x-axis shows follow-up (years). Numbers at risk are displayed at 0, 2, 4, 6, 8, and 10 years.

## 4. Discussion

In this large cohort study of 1.33 million Japanese women, our findings send a clear message: smoking is a powerful, modifiable risk factor for cervical cancer, and its detrimental effects persist even after cessation. The 47% increased risk among ever smokers, combined with a clear risk gradient across different smoking patterns (current > new ≈ former > never), provides robust epidemiological evidence supporting smoking cessation as a key pillar of cervical cancer prevention. Our findings extend previous research by assessing changes in smoking status in a large-scale Asian population, offering new insights for targeted public health initiatives.

The observed association is biologically well-supported, as smoking is known to promote carcinogenesis by impairing high-risk human papillomavirus (HPV) clearance, inducing local immunosuppression, and exerting direct mutagenic effects [10,29,30,31].

Our longitudinal results are consistent with a large body of literature linking smoking to HPV persistence and cervical neoplasia [32,33]. The hazard ratio observed in our study is comparable to findings from large-scale European and North American cohorts [5,34]. While our study’s hazard ratios reflect real-world combined effects, our findings are consistent with prior literature indicating that the association between smoking and high-grade cervical intraepithelial neoplasia and cervical cancer persists even after accounting for HPV infection status, suggesting it is unlikely to be fully explained by HPV-related confounding [12]. Crucially, this finding has been specifically validated in a meta-analysis among Japanese women [11], which strongly supports the interpretation that our results are not solely due to confounding by HPV. This study adds crucial nuance by demonstrating that newly reporting smoking (our ‘New’ category) rapidly elevates risk. This finding is significant as it suggests that regardless of this group’s precise composition—be it true initiation, relapse, or product switching—the observed change in reported status is itself a powerful marker for high risk. Furthermore, the persistently elevated risk among former smokers suggests that risk reduction after quitting is incomplete or takes a very long time [35], underscoring the critical importance of preventing smoking initiation in the first place. The attenuated risk estimate in women aged ≥65 is likely due to statistical limitations rather than a true biological difference, a common challenge in geriatric oncology epidemiology due to competing risks of mortality [36].

Given these findings, the implications for screening and prevention policies are substantial. First, our results reaffirm smoking cessation as an urgent and critical intervention alongside established primary prevention strategies like HPV vaccination and screening [37,38]. Second, our classification of dynamic smoking patterns offers a pragmatic tool to advance risk-stratified screening programs. Major public health organizations advocate for such strategies [39], and our findings provide a simple method to identify high-risk subgroups. For instance, women identified as ‘new’ or ‘persistent’ smokers could benefit from more frequent screening intervals or prioritized HPV co-testing, integrated with intensified, gender-sensitive cessation counseling.

The validity of these implications is rooted in the study’s key strengths, including its massive sample size and longitudinal design. Crucially, our case-definition strategy, which required definitive treatment codes in addition to diagnostic codes, ensures high specificity and minimizes outcome misclassification. However, our findings must be interpreted within the context of several important limitations. First, self-reported smoking status was based on a binary ‘Yes/No’ response, precluding an analysis based on smoking intensity (e.g., pack-years), duration, or product type. This is a particularly relevant limitation given Japan’s dynamic tobacco landscape, where the inability to distinguish traditional cigarettes from HTPs (due to the JMDC data structure) masks important nuances in risk [40]. Second, as noted in our methods, our ‘New’ smoker category is an operational definition and likely includes a heterogeneous mix of true initiators, relapsers and individuals switching to HTPs; the specific risks of these subgroups could not be disentangled. Third, and most importantly, the absence of data on HPV status, vaccination, and individual screening compliance means that residual confounding is possible. We could not, for example, explore the potential synergistic effect between smoking and HPV infection. Therefore, the hazard ratios should be interpreted as the combined effect of smoking and its correlated behaviors in a real-world setting, rather than the isolated biological effect of smoking alone. We were also unable to account for time-varying changes during follow-up, such as HPV vaccination status or the use of smoking cessation treatments.

Furthermore, while Japan’s 2013 HPV vaccination policy change is a critical public health event, it is unlikely to have confounded our results, as this policy primarily affected younger cohorts who are not represented in our adult, employer-based population (mean age: 45.0 years). Fifth, we lacked data on secondhand smoke exposure, which is itself an established risk factor for cervical cancer [41]. Furthermore, our cohort, derived from an employer-based insurance system, may not be fully representative of all women, potentially highlighting healthcare disparities [42,43].

To build upon our findings, future research should prioritize integrating clinical data on HPV, vaccination, and screening. Validating our claims-based algorithm and exploring smoking patterns over longer periods would also strengthen the evidence base. Ultimately, this work highlights the urgent need to integrate smoking cessation more fully into the comprehensive framework of gynecological cancer prevention. These efforts align with global health priorities, such as the WHO Framework Convention on Tobacco Control (WHO-FCTC) [44], and support the integration of smoking cessation into comprehensive cancer control programs as part of the global initiative to eliminate cervical cancer.

## 5. Conclusions

Smoking substantially increases the risk of cervical cancer. While quitting leads to a significant reduction (attenuation) of this risk compared to continued smoking, the risk for former smokers remains significantly elevated compared to those who have never smoked. These findings present a dual message: they underscore the powerful benefits of smoking cessation while also reinforcing the importance of primary prevention to avoid the long-term, residual risk.

## Figures and Tables

**Table 1:** Baseline characteristics by smoking category.

	^1^ Ever Smoker (N =173,548)			^1^ Never Smoker	^2^ *p*-Value
	Former Smoker (N = 15,929)	New Smoker (N = 10,809)	Current Smoker (N = 146,810)	(N = 1,157,249)	
Age/mean (sd)	41.3 (11.5)	39.0 (11.6)	44.4 (10.6)	43.7 (11.7)	
Age					<0.0001
<50	11,873 (74.54)	8702 (80.51)	99,947 (68.08)	790,628 (68.32)	
50–64	3721 (23.36)	1906 (17.63)	43,552 (29.67)	325,623 (28.14)	
≥65	335 (2.10)	201 (1.86)	3311 (2.26)	40,998 (3.54)	
^3^ BMI					<0.0001
Obese	745 (4.68)	470 (4.35)	7959 (5.42)	42,926 (3.71)	
Overweight	2291 (14.38)	1383 (12.79)	20,905 (14.24)	140,635 (12.15)	
Normal	10,838 (68.04)	7211 (66.71)	93,951 (63.99)	794,552 (68.66)	
Underweight	2055 (12.90)	1745 (16.14)	23,995 (16.34)	179,136 (15.48)	
Missing					
Drinking Habit					<0.0001
Daily	2715 (17.04)	2058 (19.04)	35,793 (24.38)	112,453 (9.72)	
Sometimes	5041 (31.65)	4132 (38.23)	43,151 (29.39)	379,940 (32.83)	
None	6671 (41.87)	3697 (34.20)	57,808 (39.38)	589,155 (50.91)	
Missing	1502 (9.44)	922 (8.53)	10,058 (6.85)	75,701 (6.54)	
Regular Exercise					<0.0001
Yes	2102 (13.20)	1550 (14.34)	19,669 (13.40)	97,180 (8.40)	
No	13,827 (86.80)	9259 (85.66)	127,141 (86.60)	1,060,069 (91.60)	
Comorbidities					<0.0001
Hypertension	1361 (8.54)	683 (6.32)	12,811 (8.73)	97,180 (8.40)	
Diabetes mellitus	2125 (13.34)	1187 (10.98)	16,580 (11.29)	136,993 (11.84)	
Cerebrovascular disease	878 (5.51)	458 (4.24)	5986 (4.08)	56,005 (4.84)	
Cardiovascular disease	697 (4.38)	327 (3.03)	4750 (3.24)	42,892 (3.71)	
Cholangitis	19 (0.12)	8 (0.07)	186 (0.13)	1403 (0.12)	
Year of Follow-up (Mean (sd)	3.00 ± 2.60	2.89 ± 2.40	2.9 ± 2.37	3.14 ± 2.53	<0.0001

^1^ Smoking categories (former, new, current, never) were defined based on two consecutive routine health checkups. Ever smokers include former, new, and current smokers. ^2^ *p*-values were calculated using χ^2^ tests for categorical variables and one-way ANOVA for continuous variables. ^3^ BMI categories were defined according to WHO WPRO criteria: underweight (<18.5 kg/m^2^), normal (18.5–22.9 kg/m^2^), overweight (23.0–24.9 kg/m^2^), and obese (≥25.0 kg/m^2^).

**Table 2:** Incidence rates and hazard ratios of cervical cancer by smoking status (overall and age-stratified).

				Model ^1^		Model ^2^		Model ^3^	
Group	Events	Person-Years	Incidence Rate per 100,000 Person Years (95% CI)	HR (95%CI)	*p*-Value	HR (95%CI)	*p*-Value	HR (95%CI)	*p*-Value
Total									
Never smoker	5481	3,619,696.8	151.4 (147.4–155.4)	Ref		Ref		Ref	
Ever smoker	1247	509,067.5	244.9 (231.3–258.5)	1.61 (1.52–1.72)	<0.0001	1.61 (1.52–1.72)	<0.0001	1.53 (1.43–1.62)	<0.0001
<50									
Never smoker	4130	2,510,776.3	164.4 (159.4–169.5)	Ref		Ref		Ref	
Ever smoker	993	356,055.7	278.8 (261.5–296.2)	1.69 (1.58–1.81)	<0.0001	1.67 (1.56–1.79)	<0.0001	1.58 (1.47–1.70)	<0.0001
50–64									
Never smoker	1254	1,011,613.8	123.9 (117.0–130.8)	Ref		Ref		Ref	
Ever smoker	248	144,292.2	171.8 (150.4–193.2)	1.38 (1.20–1.58)	<0.0001	1.37 (1.19–1.57)	<0.0001	1.35 (1.17–1.55)	<0.0001
≥65									
Never smoker	97	97,306.7	99.6 (79.8–119.5)	Ref		Ref		Ref	
Ever smoker	6	8719.6	68.8 (13.7–123.8)	0.69 (0.30–1.57)	0.971	0.67 (0.29–1.54)	0.963	0.69 (0.30–1.59)	0.6180

**Table 3:** Incidence rates and hazard ratios of cervical cancer by smoking category.

			Incidence Rate per 100,000 Person Years (95% CI)	Model ^1^		Model ^2^		Model ^3^	
Group	Events	Person-Years		HR (95%CI)	*p*-Value	HR (95%CI)	*p*-Value	HR (95%CI)	*p*-Value
Total									
Never smoker	5481	3,619,696.82	151.4 (147.41–155.43)	Ref		Ref		Ref	
Former smoker	109	47,579.26	229.09 (186.08–272.09)	1.51 (1.25–1.83)	<0.0001	1.52 (1.26–1.84)	<0.0001	1.44 (1.15–1.79)	<0.0001
New smoker	75	31,072.83	241.36 (186.74–295.99)	1.59 (1.27–2.00)	<0.0001	1.61 (1.28–2.02)	<0.0001	1.51 (1.20–1.90)	<0.0001
Current smoker	1063	430,415.39	246.97 (232.12–261.81)	1.63 (1.52–1.74)	<0.0001	1.63 (1.52–1.74)	<0.0001	1.54 (1.44–1.64)	<0.0001

^1^ Model 1 is univariable. ^2^ Model 2 is adjusted for age. ^3^ Model 3 is multivariable, adjusted for age, BMI category, drinking habit, regular exercise, hypertension, diabetes mellitus, cerebrovascular disease, cardiovascular disease, and cholangitis.

## Data Availability

The data presented in this study are available upon request from the corresponding author. The data are not publicly available due to privacy or ethical restrictions. The data used in this study were obtained from the Japan Medical Data Center (JMDC) claims–checkup database, which is available for research use under license. These data include de-identified health insurance claims and health checkup records and are not publicly available due to privacy protection requirements and contractual restrictions under the JMDC data use agreement. Researchers interested in accessing the JMDC database may apply for a license directly through JMDC Inc. (Tokyo, Japan). Summary-level data and analytic code supporting the findings of this study are available from the corresponding author upon reasonable request, contingent on JMDC’s data sharing policy and institutional approval.

## Abbreviations

The following abbreviations are used in this manuscript:

ANOVA	Analysis of Variance
BMI	Body Mass Index
CI	Confidence Interval
HPV	Human Papillomavirus
HR	Hazard Ratio
ICD-10	International Classification of Diseases, 10th Revision
IRB	Institutional Review Board
JMDC	Japan Medical Data Center
SD	Standard Deviation
WHO	World Health Organization
WPRO	Western Pacific Regional Office
